# Supplementary material for: MIRAGE: Robust multi-modal architectures translate fMRI-to-image models from vision to mental imagery
Source: PLoS Comput Biol. 2026 May 22;22(5):e1014263. doi: 10.1371/journal.pcbi.1014263 (PMC13218618; doi:10.1371/journal.pcbi.1014263)
Supplement: S1 Text — Fig A: Hyperparameter logarithmic grid search over possible values of λ for use in Equation 1 (Section 4.2.3). Metrics are the normalized average of all metrics in Table 1 of the manuscript, with imagery performance on the Y axis and vision on the X axis. Fig B: Qualitative comparison of reconstruction methods on stimuli seen during the vision trials of NSD-Imagery. Samples selected are the best scoring according to the reconstruction metrics in Table 1 of the manuscript. Fig C: Median-case vision reconstructions from the vision trials of NSD-Imagery. Samples selected as median scoring based on metrics in Table 1 of the manuscript. Fig D: Median-case imagery reconstructions from the imagery trials of NSD-Imagery. Samples selected as in Fig C. Fig E: Worst-case vision reconstructions from the vision trials of NSD-Imagery. Samples selected as lowest scoring based on metrics in Table 1 of the manuscript. Fig F: Worst-case imagery reconstructions from the imagery trials of NSD-Imagery. Samples selected as in Fig E. Fig G: Best-case vision reconstructions (additional methods) from vision trials of NSD-Imagery. Samples selected as highest scoring based on metrics in Table 1 of the manuscript. Fig H: Best-case imagery reconstructions (additional methods) from imagery trials of NSD-Imagery. Samples selected as in Fig G. Fig I: Median-case vision reconstructions (additional methods) from vision trials of NSD-Imagery. Samples selected as median scoring based on metrics in Table 1 of the manuscript. Fig J: Median-case imagery reconstructions (additional methods) from imagery trials of NSD-Imagery. Samples selected as in Fig I. Fig K: Worst-case vision reconstructions (additional methods) from vision trials of NSD-Imagery. Samples selected as lowest scoring based on metrics in Table 1 of the manuscript. Fig L: Worst-case imagery reconstructions (additional methods) from imagery trials of NSD-Imagery. Samples selected as in Fig K. Table A: Standard error measurements for evaluat [file pcbi.1014263.s001.pdf]

A Appendix

A.1 Finding the optimal value of λ for the regression backbone

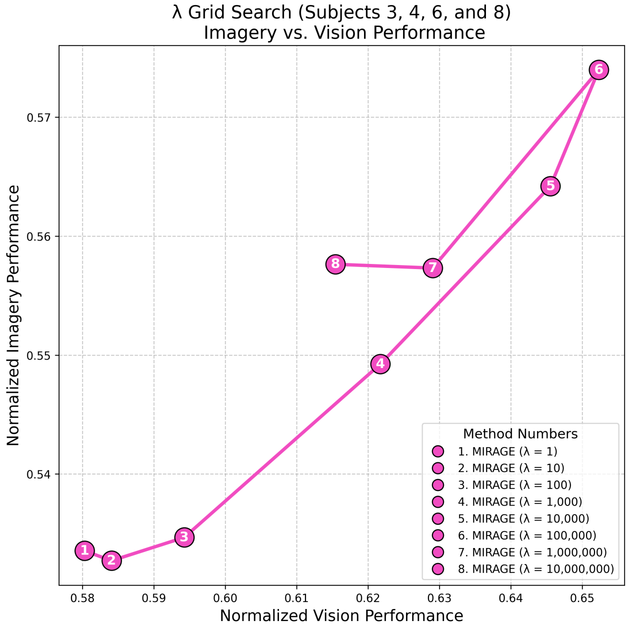

Figure A: Hyperparameter logarithmic grid search over possible values of λ for use in Equation 1 (Section 4.2.3). Metrics are the normalized average of all metrics in Table 1 of the manuscript, with imagery performance on the Y axis and vision on the X axis.

To select the optimal L_2 weight decay parameter λ for use in our regression backbone detailed in Equation 1 of Section 4.2.3, we performed a logarithmic grid search over possible values of λ on subjects $3, 4, 6$, and 8 of the NSD-Imagery dataset, which we use a hyperparameter tuning set. From this analysis (Fig A), we select $\lambda = 100,000$.

A.2 VDVAE implementation

For decoding the low-level image in our pipeline, we utilize the Very Deep Variational Autoencoder (VDVAE) model introduced in Child [37]. VDVAEs are generative models that learn to represent an input distribution—such as an image dataset—through a low-dimensional latent space constrained by a predefined prior distribution, typically Gaussian. The VDVAE utilizes a hierarchical structure with multiple layers of conditionally dependent latent variables organized hierarchically, with each layer capturing different levels of detail from coarse to fine as one moves from the top to the bottom of the hierarchy. Appendix Eq. (2) shows the factorization of the variational posterior, where each latent z_n corresponds to one layer of the VDVAE. Eq. (3) shows the factorization of the prior.

$$q_\phi(z|x) = q_\phi(z_0|x)q_\phi(z_1|z_0, x)...q_\phi(z_N|z_{<N}, x) \tag{2}$$
$$p_\theta(z) = p_\theta(z_0)p_\theta(z_1|z_0)...p_\theta(z_N|z_{<N}) \tag{3}$$

In our approach, we utilize the VDVAE model [37] trained on the ImageNet dataset at a resolution of 64×64 pixels and consisting of 75 hierarchical layers. We use the latent variables from the first 31 layers, as including additional layers does not yield significant improvements in reconstruction quality. In the testing phase, our predicted latents for the first 31 layers are concatenated with the remaining 44 layers sampled from Eq. (2), and passed through the latent-to-pixel decoder module of the VDVAE to generate reconstructed images at 64×64 pixel resolution.

Following the generation of these initial reconstructions, we apply a post-processing step to enhance their visual clarity using the `PIL.ImageEnhance` module in Python. Specifically, we boost the sharpness and contrast of the low-level images using the following implementation:

```
blurred_image = ImageEnhance.Sharpness(blurred_image).enhance(20)
blurred_image = ImageEnhance.Contrast(blurred_image).enhance(1.5)
```

We did not perform a formal hyperparameter optimization for these specific values; rather, they were selected heuristically. These parameters were chosen because they qualitatively produced the desired visual corrections to the low-level images when testing the pipeline on the held-out NSD subjects (Subjects 3, 4, 6, and 8) that were not included in the main evaluation.

A.3 Best case vision reconstructions

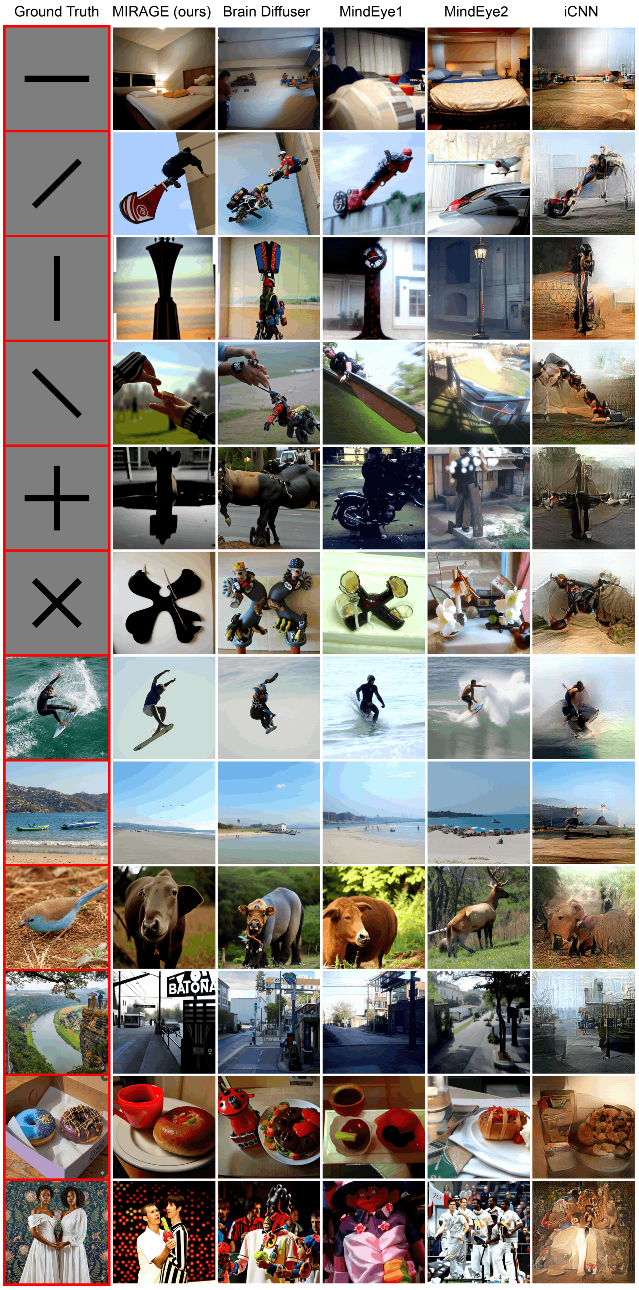

Figure B: Qualitative comparison of reconstruction methods on stimuli seen during the vision trials of NSD-Imagery. Samples selected are the best scoring according to the reconstruction metrics in Table 1 of the manuscript.

A.4 Median and worst-case reconstructions

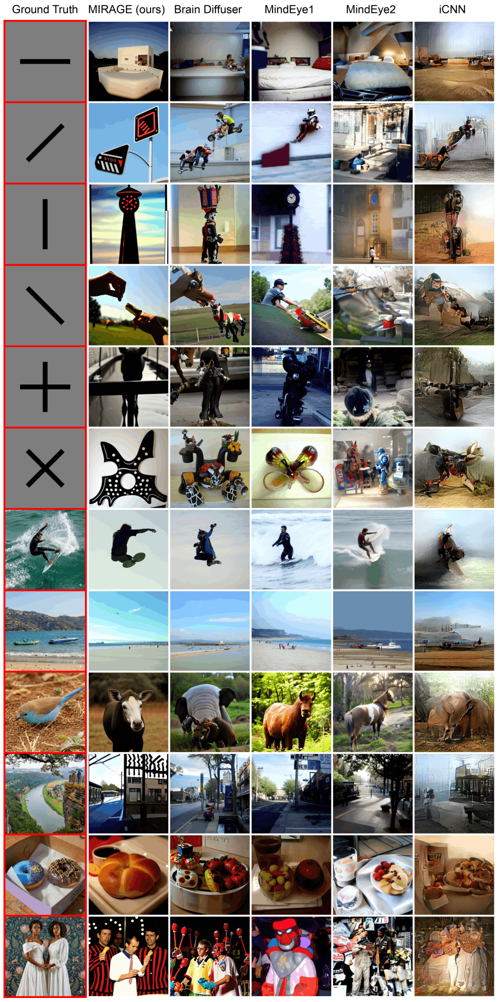

Figure C: Median-case vision reconstructions from the vision trials of NSD-Imagery. Samples selected as median scoring based on metrics in Table 1 of the manuscript.

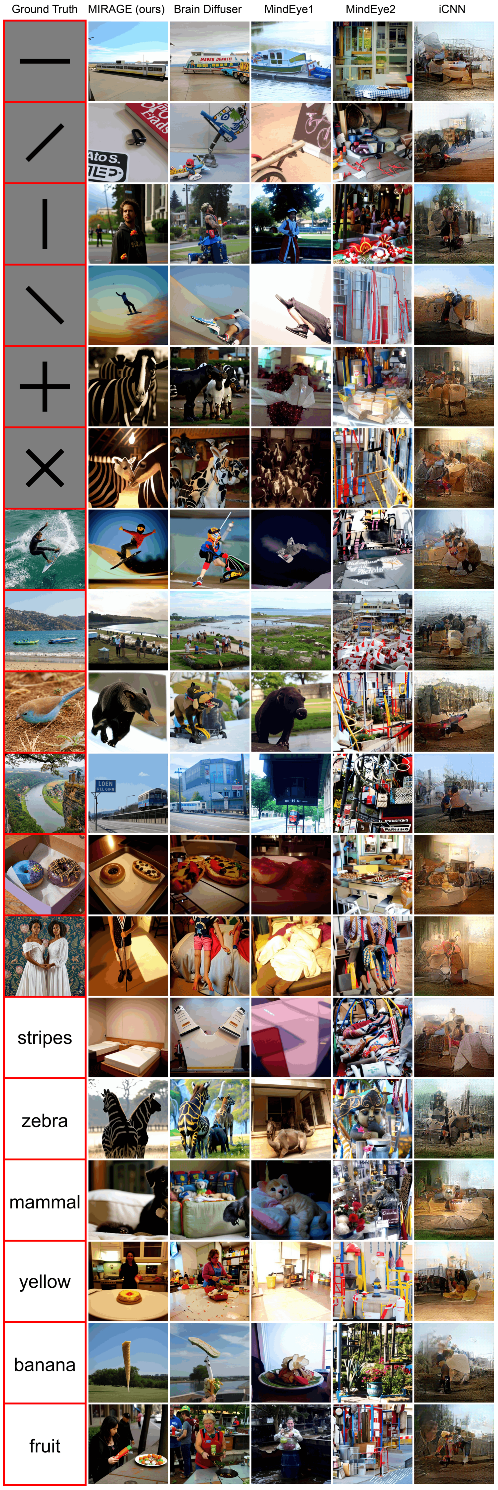

Figure D: Median-case imagery reconstructions from the imagery trials of NSD-Imagery. Samples selected as in Fig C.

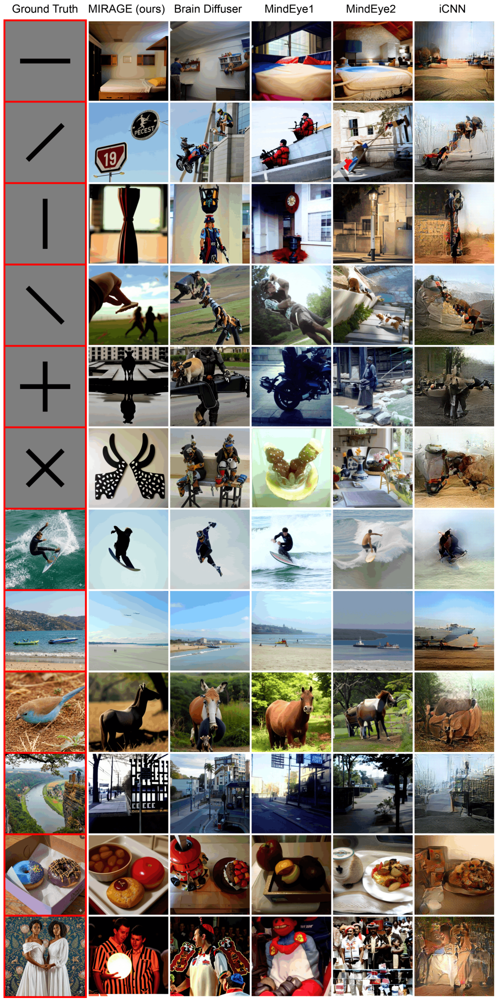

Figure E: Worst-case vision reconstructions from the vision trials of NSD-Imagery. Samples selected as lowest scoring based on metrics in Table 1 of the manuscript.

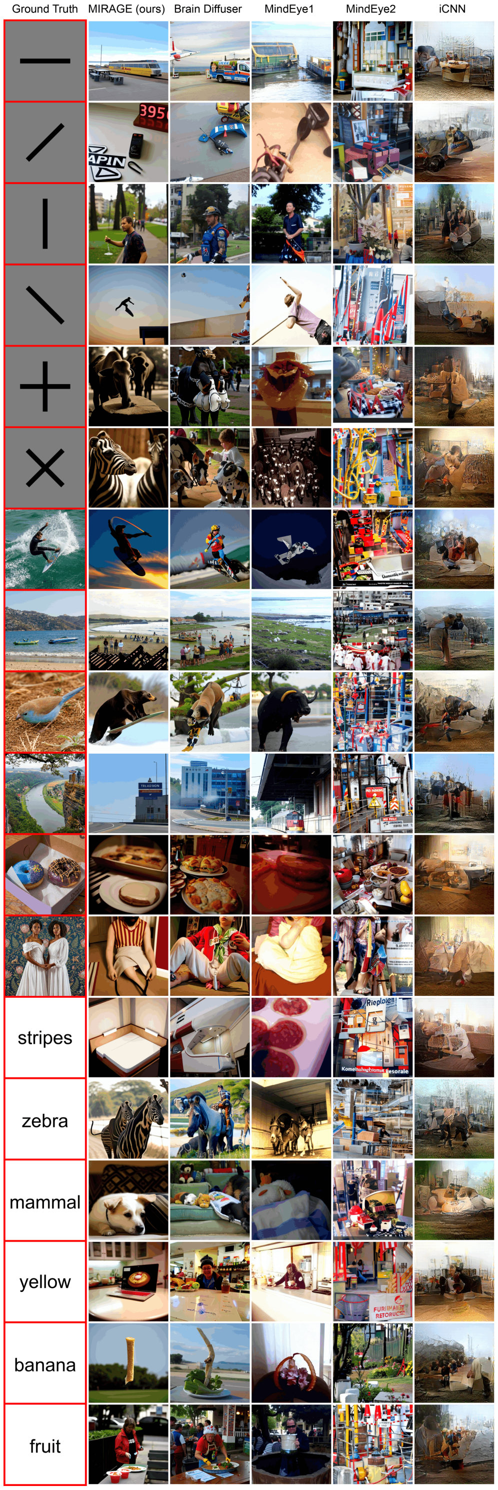

Figure F: Worst-case imagery reconstructions from the imagery trials of NSD-Imagery. Samples selected as in Fig E.

A.5 Reconstructions from additional methods on NSD-Imagery

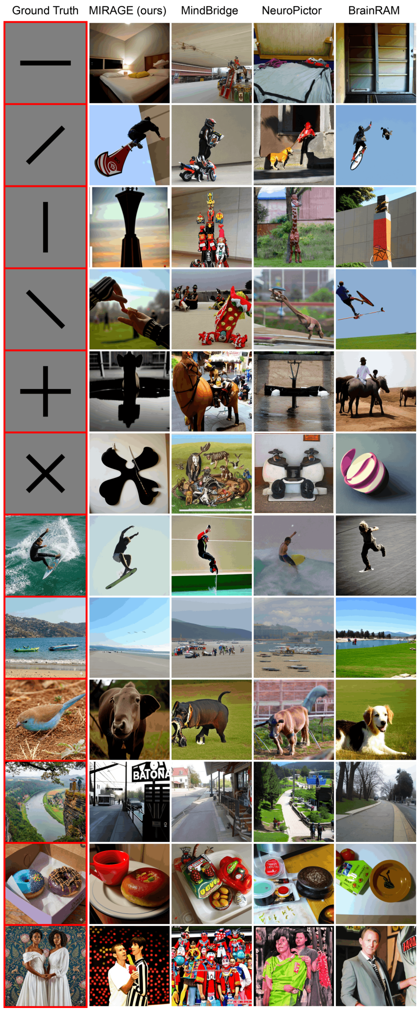

Figure G: Best-case vision reconstructions (additional methods) from vision trials of NSD-Imagery. Samples selected as highest scoring based on metrics in Table 1 of the manuscript.

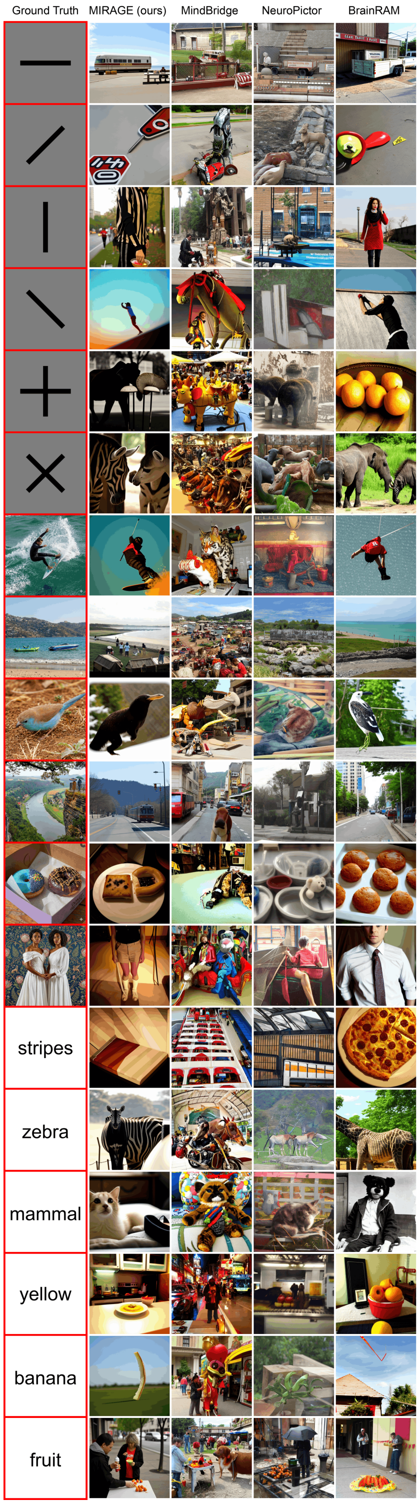

Figure H: Best-case imagery reconstructions (additional methods) from imagery trials of NSD-Imagery. Samples selected as in Fig G.

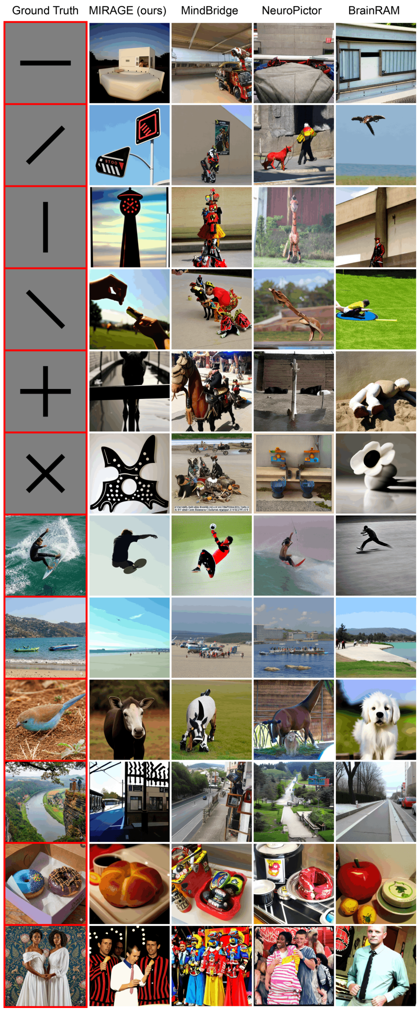

Figure I: Median-case vision reconstructions (additional methods) from vision trials of NSD-Imagery. Samples selected as median scoring based on metrics in Table 1 of the manuscript.

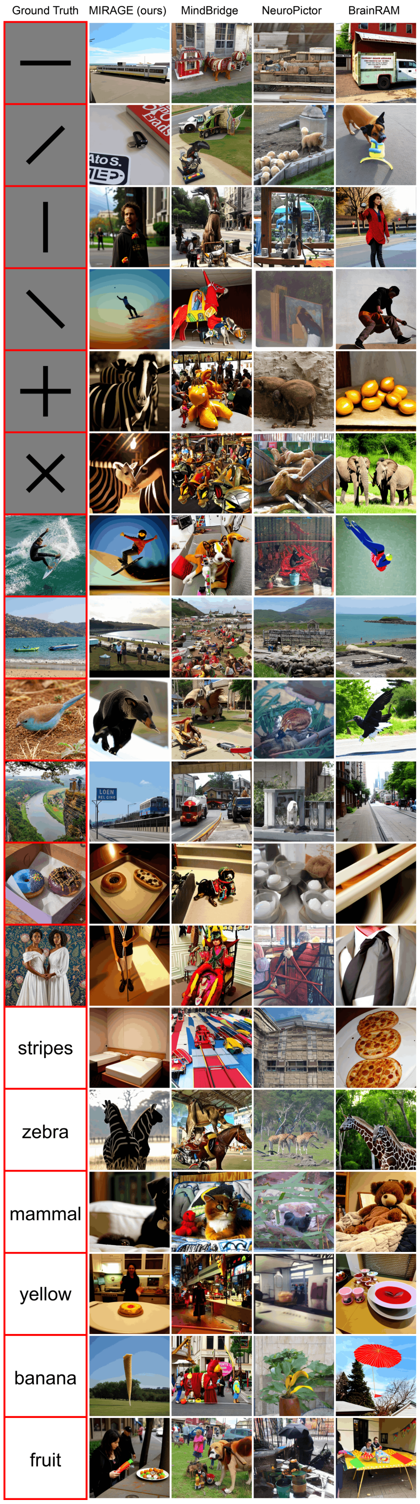

Figure J: Median-case imagery reconstructions (additional methods) from imagery trials of NSD-Imagery. Samples selected as in Fig I.

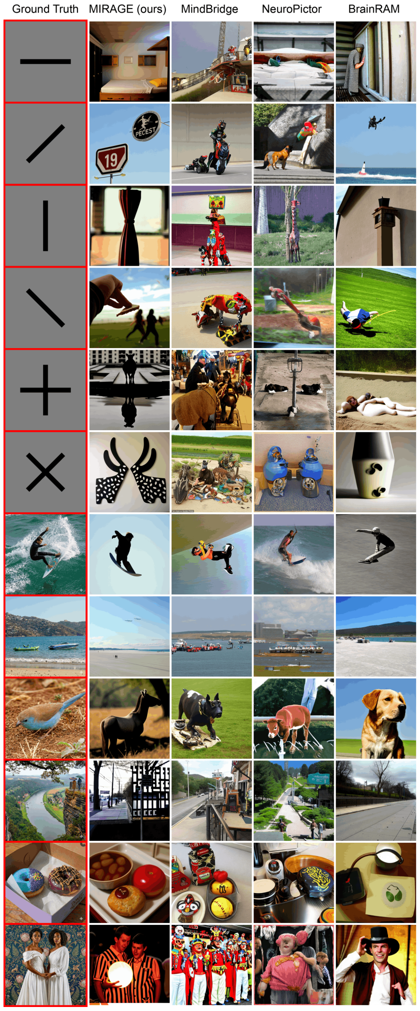

Figure K: Worst-case vision reconstructions (additional methods) from vision trials of NSD-Imagery. Samples selected as lowest scoring based on metrics in Table 1 of the manuscript.

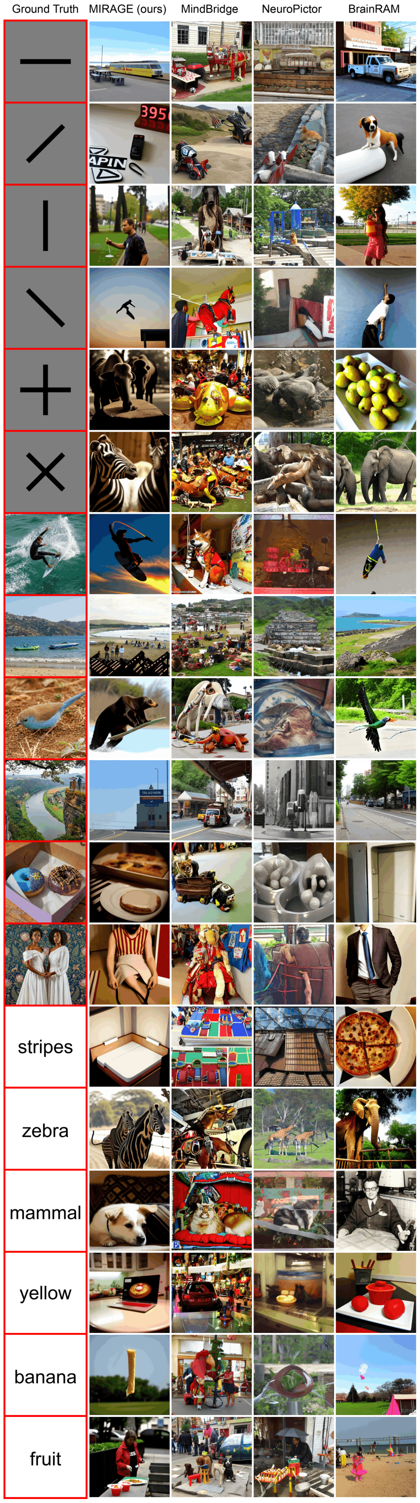

Figure L: Worst-case imagery reconstructions (additional methods) from imagery trials of NSD-Imagery. Samples selected as in Fig K.

A.6 Additional evaluation metric details

For the metrics in Table 1 of the manuscript, a two-way comparison evaluates whether the feature embedding of the stimulus image is more similar to the feature embedding of the target reconstruction, or the feature embedding of a randomly selected "distractor" reconstruction. Two-way identification refers to percent correct across a set of two-way comparisons performed on a pool of distractor images. The two-way identification metrics we report, which are calculated using reconstructions of the 11 other NSD-Imagery stimuli as distractors, are notably different from the two-way identification metrics presented in individual reconstruction papers that perform evaluations using reconstructions of the shared1000 as the pool of distractors. The pool of distractor images for NSD-Imagery is much smaller, and contains multiple distinct types of stimuli that may significantly alter the resulting identification accuracy metrics. Because of this difference, the two-way identification accuracy numbers are not directly comparable to two-way identification results evaluated on the shared1000 in our work or in other papers. Brain correlation scores are the Pearson correlation between the averaged measured brain response β and the predicted brain response β' produced by a brain encoding model (GNet [72]) averaged across voxels within a respective ROI in visual cortex, including the whole visual cortex, early visual cortical regions (V1, V2, V3, and V4), and higher visual areas (set complement of visual cortex and early visual cortex). All metrics in Tables 1, Appendix Table C, and Appendix TableD were calculated and averaged across 10 images sampled from the output distribution of each method using a random seed. The caption metrics were computed against the ground truth image captions provided with the NSD-Imagery dataset. Metrics in Table 1 of the manuscript are the original values reported in each of the respective papers, except for the iCNN method, which has never been benchmarked on the NSD shared1000 test set and so results reported are from our reproduction of the method utilizing the author's open source code. For the results from our method (MIRAGE) in the table, we compute values across 5 output repetitions sampled from the posterior of our method, and average those values together for the table.

A.6.1 Normalized average of image feature metrics

To compute the normalized average of the image feature metrics used in Fig 4B and to select the best, median, and worst reconstructions displayed in figures throughout our paper, we first standardized each metric S_k to the unit interval $[0, 1]$. For metrics where lower values indicated better performance (denoted by the set $\mathcal{L}$), we applied an inverted Min-Max normalization such that the optimal raw value mapped to 1. The final score $\bar{S}_{\text{final}}$ was computed as the arithmetic mean of these normalized values, ensuring equal weighting across all image feature metrics:

$$\bar{S}_{\text{final}} = \frac{1}{K} \sum_{k=1}^{K} \left(\begin{cases} \frac{\max(S_k) - S_k}{\max(S_k) - \min(S_k)} & \text{if } k \in \mathcal{L} \\ \frac{S_k - \min(S_k)}{\max(S_k) - \min(S_k)} & \text{otherwise} \end{cases} \right) \tag{4}$$

Where:

- K is the total number of metrics.
- S_k denotes the raw score of the k-th metric.
- $\mathcal{L}$ is the set of metrics where a lower score indicates better performance.
- $\min(S_k)$ and $\max(S_k)$ are the minimum and maximum values of metric k across the dataset.

A.7 Statistical significance of metrics

Method	Low-Level				High-Level				Brain Correlation		
	PixCorr ↑	SSIM ↑	Alex(2) ↑	Alex(5) ↑	Incep ↑	CLIP ↑	Eff ↓	SwAV ↓	Early Vis. ↑	Higher Vis. ↑	Visual Cortex ↑
**Mental Imagery Reconstructions**											
MIRAGE	±0.0061	±0.0091	±0.89%	±1.06%	±1.27%	±1.24%	±0.0044	±0.0040	±0.0075	±0.0067	±0.0059
MindEye1	±0.0082	±0.0082	±1.07%	±0.72%	±1.43%	±1.35%	±0.0064	±0.0047	±0.0073	±0.0075	±0.0069
Brain Diffuser	±0.0068	±0.0087	±1.40%	±1.07%	±1.47%	±1.46%	±0.0053	±0.0041	±0.0086	±0.0079	±0.0070
iCNN	±0.0081	±0.0055	±0.93%	±0.54%	±1.42%	±1.21%	±0.0041	±0.0023	±0.0055	±0.0074	±0.0056
MindEye2	±0.0085	±0.0079	±1.07%	±0.87%	±1.41%	±1.27%	±0.0067	±0.0051	±0.0073	±0.0081	±0.0070
MindBridge	±0.0063	±0.0080	±1.38%	±1.11%	±1.45%	±1.24%	±0.0053	±0.0045	±0.0079	±0.0079	±0.0071
NeuroPictor	±0.0062	±0.0072	±1.23%	±1.22%	±1.53%	±1.37%	±0.0046	±0.0037	±0.0087	±0.0080	±0.0072
BrainRAM	±0.0074	±0.0105	±1.30%	±1.21%	±1.49%	±1.26%	±0.0058	±0.0056	±0.0088	±0.0074	±0.0074
**Vision Reconstructions**											
MIRAGE	±0.0072	±0.0097	±1.23%	±1.21%	±1.56%	±1.41%	±0.0040	±0.0045	±0.0052	±0.0050	±0.0044
MindEye1	±0.0086	±0.0086	±1.35%	±1.31%	±1.53%	±1.53%	±0.0035	±0.0036	±0.0053	±0.0046	±0.0042
Brain Diffuser	±0.0052	±0.0082	±1.38%	±1.35%	±1.49%	±1.50%	±0.0040	±0.0038	±0.0055	±0.0051	±0.0045
iCNN	±0.0077	±0.0052	±1.24%	±1.26%	±1.40%	±1.40%	±0.0021	±0.0025	±0.0058	±0.0052	±0.0044
MindEye2	±0.0049	±0.0084	±1.45%	±1.43%	±1.60%	±1.51%	±0.0034	±0.0037	±0.0054	±0.0053	±0.0048
MindBridge	±0.0046	±0.0041	±1.43%	±1.48%	±1.54%	±1.48%	±0.0029	±0.0036	±0.0064	±0.0048	±0.0047
NeuroPictor	±0.0046	±0.0060	±1.40%	±1.54%	±1.47%	±1.44%	±0.0021	±0.0028	±0.0053	±0.0053	±0.0048
BrainRAM	±0.0063	±0.0087	±1.48%	±1.36%	±1.49%	±1.43%	±0.0044	±0.0047	±0.0053	±0.0048	±0.0044

Table A: Standard error measurements for evaluation metrics of fMRI-to-Image reconstruction models evaluated on both the vision and mental imagery trials of NSD-Imagery. Values correspond to the standard error spread of values in Table 1 in the manuscript.

A.8 NSD test set feature metric evaluations

**NSD Shared1000 Test Set**	Low-Level				High-Level				Brain Correlation		
Method	PixCorr ↑	SSIM ↑	Alex(2) ↑	Alex(5) ↑	Incep ↑	CLIP ↑	Eff ↓	SwAV ↓	Early Vis. ↑	Higher Vis. ↑	Visual Cortex ↑
**MIRAGE (ours)**	0.285	0.361	94.30%	95.73%	91.18%	90.92%	0.732	0.473	0.337	0.371	0.372
**MindEye1 [29]**	0.319	0.360	92.49%	96.44%	93.55%	92.14%	0.648	0.377	0.350	0.374	0.378
**Brain Diffuser [28]**	0.273	0.365	94.39%	96.64%	91.28%	90.90%	0.728	0.421	0.353	**0.375**	0.381
**iCNN [21]**	0.321	0.336	94.33%	97.09%	90.46%	74.47%	0.797	0.528	**0.410**	0.371	**0.396**
**MindEye2 [1]**	**0.322**	**0.431**	**96.10%**	**98.61%**	**95.42%**	**92.98%**	**0.619**	**0.344**	0.360	0.368	0.373

Table B: Quantitative comparison between reconstruction methods on the NSD Shared1000 Test Set. Metrics are the same as Table 1 of the manuscript.

A.9 Comparison of image feature metrics across stimuli types

Method	Low-Level				High-Level				Brain Correlation		
	PixCorr ↑	SSIM ↑	Alex(2) ↑	Alex(5) ↑	Incep ↑	CLIP ↑	Eff ↓	SwAV ↓	Early Vis. ↑	Higher Vis. ↑	Visual Cortex ↑
**Mental Imagery Reconstructions (Simple Stimuli)**											
**MIRAGE (ours)**	0.027	0.511	**53.11%**	**67.27%**	42.39%	60.30%	**0.939**	0.563	**0.224**	**0.118**	**0.164**
**MindEye1 [29]**	0.033	0.456	43.71%	61.67%	37.46%	58.37%	0.974	0.563	0.200	0.107	0.148
**Brain Diffuser [28]**	0.013	**0.524**	30.68%	50.68%	34.43%	44.51%	0.983	0.603	0.152	0.091	0.128
**iCNN [21]**	**0.063**	0.427	27.42%	47.65%	**45.11%**	**67.99%**	1.006	**0.546**	0.138	0.045	0.081
**MindEye2 [1]**	0.011	0.448	23.37%	45.34%	31.14%	49.02%	0.987	0.590	0.074	0.035	0.051
**Vision Reconstructions (Simple Stimuli)**											
**MIRAGE (ours)**	**0.159**	0.569	**74.24%**	**82.77%**	**56.78%**	63.71%	**0.913**	0.537	0.395	**0.174**	0.279
**MindEye1 [29]**	0.129	0.506	62.01%	76.36%	43.33%	60.64%	0.961	0.549	0.370	0.140	0.243
**Brain Diffuser [28]**	0.075	**0.586**	40.19%	66.67%	38.30%	42.20%	0.988	0.601	0.209	0.106	0.169
**iCNN [21]**	0.132	0.454	57.01%	74.89%	37.69%	**69.02%**	0.992	**0.534**	**0.447**	0.133	**0.278**
**MindEye2 [1]**	0.040	0.487	50.87%	68.98%	43.52%	52.46%	0.980	0.577	0.334	0.108	0.204

Table C: Quantitative comparison between reconstruction methods for both imagery and vision trials on simple stimuli. Metrics are the same as Table 1 of the manuscript.

Method	Low-Level				High-Level				Brain Correlation		
	PixCorr ↑	SSIM ↑	Alex(2) ↑	Alex(5) ↑	Incep ↑	CLIP ↑	Eff ↓	SwAV ↓	Early Vis. ↑	Higher Vis. ↑	Visual Cortex ↑
**Mental Imagery Reconstructions (Complex Stimuli)**											
**MIRAGE (ours)**	**0.181**	0.285	74.74%	57.65%	62.121%	54.62%	0.888	0.587	**0.183**	**0.165**	**0.172**
**MindEye1 [29]**	0.138	0.243	**75.42%**	60.34%	66.591%	51.06%	0.921	**0.566**	0.159	0.164	0.161
**Brain Diffuser [28]**	0.114	**0.278**	73.60%	**66.02%**	**71.02%**	**63.64%**	**0.888**	0.567	0.114	0.163	0.154
**iCNN [21]**	0.153	0.253	73.71%	62.84%	53.674%	15.46%	0.982	0.575	0.089	0.079	0.081
**MindEye2 [1]**	0.032	0.231	70.42%	65.11%	61.97%	51.93%	0.943	0.601	0.062	0.074	0.068
**Vision Reconstructions (Complex Stimuli)**											
**MIRAGE (ours)**	0.282	0.315	83.83%	70.38%	82.727%	69.659%	0.845	0.555	0.331	0.350	0.353
**MindEye1 [29]**	0.308	0.318	85.11%	85.27%	81.55%	70.038%	0.800	0.471	0.378	0.365	0.379
**Brain Diffuser [28]**	0.139	**0.323**	80.49%	79.02%	83.60%	74.43%	0.829	0.509	0.284	0.353	0.341
**iCNN [21]**	**0.316**	0.316	**86.33%**	**87.80%**	**84.62%**	29.05%	0.860	0.514	**0.437**	0.358	**0.397**
**MindEye2 [1]**	0.223	0.333	84.28%	85.83%	80.08%	**77.46%**	**0.794**	**0.454**	0.378	**0.360**	0.376

Table D: Quantitative comparison between reconstruction methods for both imagery and vision trials on complex stimuli. Metrics are the same as Table 1 of the manuscript.

A.10 Impact of trial repetition averaging on performance

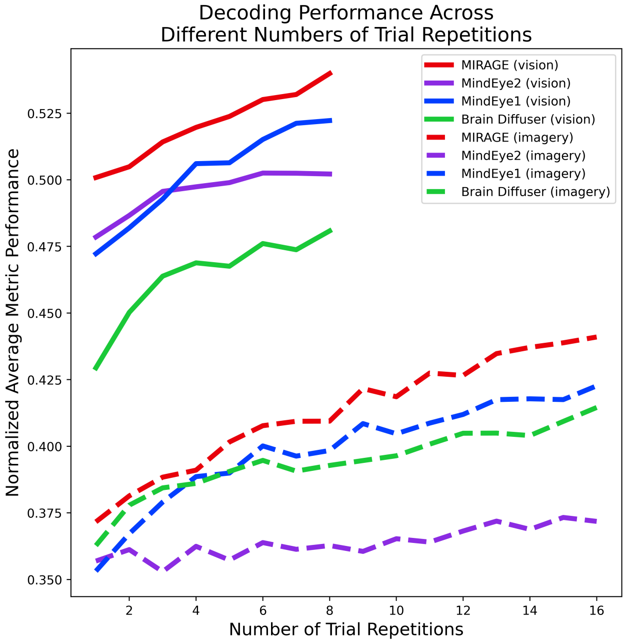

Figure M: Performance of **MIRAGE** and other methods when averaging across brain activity responses to multiple trial repetitions of the same stimulus. Y-axis is the normalized average of all metrics in Table 1 of the manuscript, X-axis is the number of averaged trial repetitions.

One of the experimental details that varies between NSD [25] and NSD-Imagery [2] is the number of times each stimulus was presented in the experiment, also called the number of trial repetitions. NSD contained 3 trial repetitions of each stimulus in both the training and test sets, while NSD-Imagery contains 8 trial repetitions for the vision task and 16 trial repetitions for the imagery task. In Fig M, we plot the effect of these additional trial repetitions on the performance of **MIRAGE** relative to the other methods we compare against.

A.11 Impact of training data scale on performance

An additional challenge in deploying these fMRI-to-image decoding methods lies in making them more generalizable to new subjects. **MIRAGE**, along with all of the other methods examined in this paper, were trained with 40 hours of subject-specific fMRI data comprising 10,000 unique stimuli. Collecting this much training data for new subjects in practical settings is currently impractical or

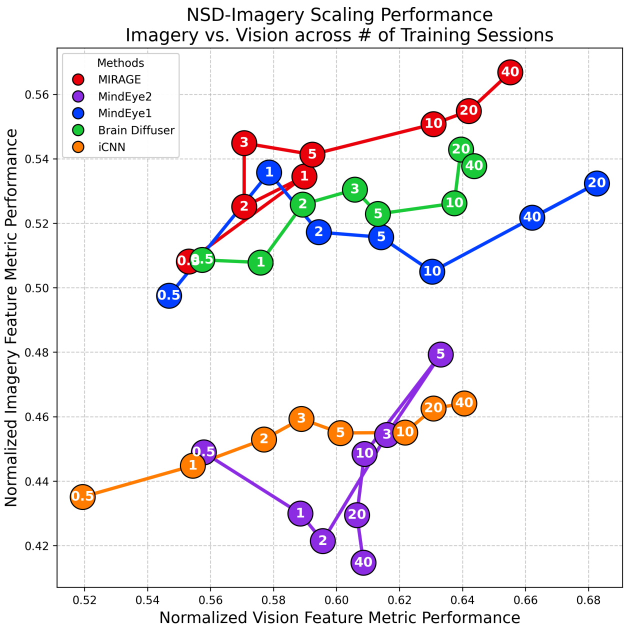

Figure N: Performance of **MIRAGE** and other methods on NSD-Imagery for Subject 1 when trained on different numbers of fMRI sessions present in NSD. Each session includes approximately one hour of fMRI data. Metrics are the normalized average of all metrics in Table 1 of the manuscript, with imagery performance on the Y axis and vision on the X axis. Methods are indicated by color, with the number of training sessions indicated by the numbers in each dot.

impossible for certain clinical patients. Recent work in MindEye2 [1] has tackled this problem head-on by using a multi-subject pretraining step, however as evaluated in Fig N, this technique generalizes poorly to mental imagery data. By contrast, MIRAGE outperforms all other methods for mental image reconstruction using only 3 hours of fMRI training data, and continues to scale robustly up to 40 sessions. We additionally note that the methods that used ridge regression decoding backbones (**MIRAGE**, Brain Diffuser, iCNN) all produce much more consistent scaling improvements on mental images than the models that utilize deep neural network backbones (MindEye1, MindEye2).

A.12 Impact of diffusion strength on performance

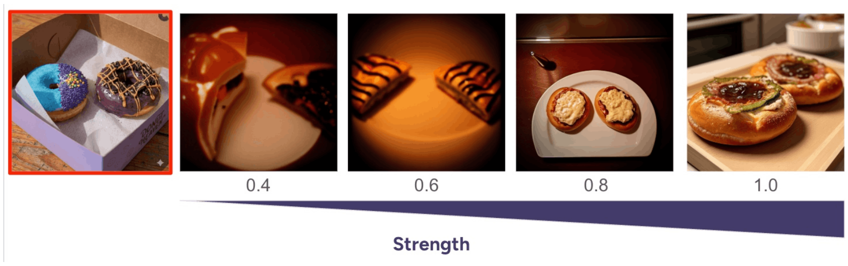

Figure O: Examples of reconstructions provided at different diffusion strength parameters, images are the ground truth (outlined in red) and reconstructions provided at 0.4, 0.6, 0.8, and 1.0 diffusion strength respectively. Strength values below 0.4 experience no noticeable variation due to the nonlinear dynamics of the strength parameter.

Recent work [51, 52] has raised the question of how much of the detail in fMRI-to-image reconstructions originates in the brain and how much is simply hallucinated by the strong natural priors enforced by a diffusion model. This critical perspective makes a clear prediction: As the strength of the natural prior increases, the results should improve. One way of examining this relationship is by

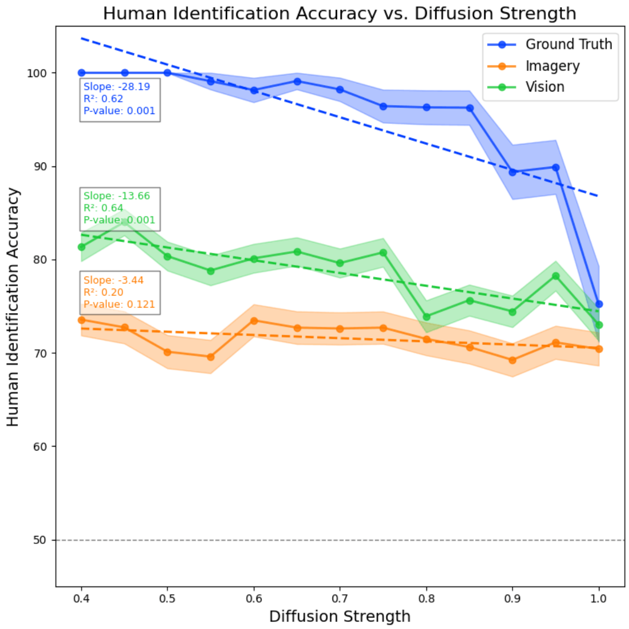

Figure P: Human identification accuracy of **MIRAGE** (with no CLIP-Image guidance) as a function of diffusion model strength for imagery trials (orange line), vision trials (green line), and a control experiment that used the features directly from the ground truth image and caption (blue line). A dashed line is placed at the 50% chance threshold. Results are from a behavioral experiment that is identical to Experiment 1 (Fig 3A), but varied across strength parameters.

modulating the strength parameter in the img2img mode of the diffusion model, by which an initial image (in our case the low-level reconstruction provided by the VDVAE model discussed in Section 4.2.4) is partially noised and then denoised with CLIP guidance. This denoising process is where the natural priors are enforced, and the amount of denoising (and thus the amount of the final image that is guided by the natural prior) is modulated by the strength parameter. We repeated Experiment 1 presented in Fig 3A of the paper across a wide range of strength parameters to investigate the potential influence of diffusion strength on the results. We test strength parameters between 0.4—the lowest strength value that yields meaningful variation from the input image—and 1.0, which destroys the entire input image before denoising. In this experiment, we use only CLIP-text semantic guidance for the diffusion process to increase the contrast between the original input image and the purely semantic guidance during the denoising process, although we acknowledge that this slightly reduces the performance of **MIRAGE** during the experiment relative to the results in Fig 3A. The results demonstrate that increasing the strength parameter (and therefore increasing the influence of the prior in determining the ultimate reconstruction) induces no significant change in the rate at which humans can correctly identify mental image reconstructions as corresponding to the stimulus image, and seen image reconstructions experience a significant *decrease* in identifiability as diffusion strength increases (Fig P). Although the diffusion model prior clearly plays a role in improving the quality and aesthetics of the reconstructions, our results show that there is still plenty of decoded signal present in the reconstructions to facilitate identification even with minimal guidance from the diffusion model.

To further emphasize our improved signal recovery and decreased reliance on the priors of the image generator, MIRAGE employs the lowest diffusion strength parameter (0.7) among comparable "dual stream" (high-level/low-level) reconstruction methods, including as Brain Diffuser (0.75) and MindEye1 (0.85), meaning MIRAGE explicitly relies less on the diffusion model's prior than other approaches. Additionally, MIRAGE achieves state-of-the-art (SOTA) performance on both simple and conceptual stimuli types, which are outside the natural prior of the diffusion model. These findings underscore MIRAGE's ability to extract and utilize more imagery signal from the brain independent of the natural prior of the diffusion model, setting it apart from alternative methods.

A.13 Retrieval analysis

To assess the necessity of MIRAGE's generative complexity, we compared our method against a baseline of direct image retrieval. Fundamentally, retrieval and reconstruction differ in the nature of their image priors: retrieval relies on a static image corpus, effectively acting as a prior that assigns a non-zero, uniform probability to a finite set of candidate images and zero probability to all other possible images. In contrast, reconstruction utilizes a diffusion model, which models a continuous probability distribution across the broader manifold of natural images from its training distribution. While a systematic comparison exploring the interaction between various decoded feature spaces and retrieval corpus distributions is beyond the scope of this work, we evaluated a targeted baseline to determine if the diffusion model provides distinct value over retrieval within MIRAGE's selected feature spaces and the shared1000 retrieval pool commonly used in other work [1, 29].

We performed retrieval in the pooled ViT-L/14 image embedding space used to drive the MIRAGE generative model, and the hidden layer ViT-L/14 space utilized in the retrieval pooling step (Section 4.2.6) using a candidate corpus of 1000 COCO [61] images sourced from the NSD shared1000. As demonstrated in Table E, MIRAGE substantially outperforms all retrieval baselines on most high-level and semantic metrics, while hidden-layer retrieval remains competitive on certain low-level metrics. These results confirm that appending a diffusion model to the end of the MIRAGE pipeline yields fundamentally superior, bespoke image decoding performance compared to selecting likely approximations from a static corpus.

Method	Low-Level				High-Level				Brain Correlation		
	PixCorr ↑	SSIM ↑	Alex(2) ↑	Alex(5) ↑	Incep ↑	CLIP ↑	Eff ↓	SwAV ↓	Early Vis. ↑	Higher Vis. ↑	Visual Cortex ↑
**Mental Imagery Reconstructions (Simple Stimuli)**											
**MIRAGE (ours)**	**0.027**	0.511	**53.11%**	**67.27%**	**42.39%**	**60.30%**	0.939	**0.563**	**0.224**	**0.118**	**0.164**
Top-1 Retrieval	-0.022	0.446	28.79%	42.42%	36.36%	53.03%	1.007	0.603	0.053	0.099	0.105
Top-1 Retrieval (hidden layer)	0.015	**0.531**	33.33%	51.52%	31.82%	48.48%	**0.880**	0.596	0.136	0.116	0.145
**Vision Reconstructions (Simple Stimuli)**											
**MIRAGE (ours)**	**0.159**	0.569	**74.24%**	**82.77%**	**56.78%**	**63.71%**	0.913	0.537	**0.395**	**0.174**	**0.279**
Top-1 Retrieval	-0.062	0.500	12.12%	46.97%	37.88%	48.48%	0.971	0.602	0.080	0.067	0.111
Top-1 Retrieval (hidden layer)	0.034	**0.640**	42.42%	56.06%	54.18%	62.18%	**0.730**	**0.430**	0.134	0.127	0.170
**Mental Imagery Reconstructions (Complex Stimuli)**											
**MIRAGE (ours)**	**0.181**	0.285	**74.74%**	**57.65%**	**62.12%**	54.62%	0.888	0.587	**0.183**	**0.165**	**0.172**
Top-1 Retrieval	0.090	0.229	63.64%	56.09%	45.45%	**63.64%**	0.952	0.601	0.181	0.065	0.137
Top-1 Retrieval (hidden layer)	0.153	**0.352**	43.94%	34.85%	37.88%	48.48%	**0.756**	**0.480**	0.164	0.111	0.139
**Vision Reconstructions (Complex Stimuli)**											
**MIRAGE (ours)**	**0.282**	0.315	**83.83%**	**70.38%**	**82.73%**	**69.66%**	0.845	0.555	**0.331**	**0.350**	**0.353**
Top-1 Retrieval	0.196	0.294	78.79%	68.18%	53.03%	56.06%	0.866	0.541	-0.016	0.238	0.150
Top-1 Retrieval (hidden layer)	0.179	**0.382**	69.70%	54.55%	81.33%	34.85%	**0.716**	**0.447**	0.187	0.235	0.231

Table E: Quantitative comparison between MIRAGE and two Top-1 Retrieval baselines (pooled and hidden layer CLIP ViT-L/14 embeddings), separated by simple and complex stimuli and averaged across all subjects. Metrics are the same as Table 1. Bold indicates the best performance between MIRAGE and the retrieval baselines within each stimulus category, and underlines indicate second-best.

We also evaluated the dynamics of a set of common "Top-K" retrieval metrics (top-1, top-5, and top-10) as a function of the size of the retrieval pool being used (Fig Q). Since these metrics measure the reliability of being able to extract the exact stimulus image from a pool, we added the set of NSD-Imagery stimulus images to our retrieval pool for this analysis, while noting that these stimuli are often out of distribution for this particular retrieval pool. Predictably, retrieval accuracy when using the pooled CLIP ViT-L/14 embeddings fails for simple stimuli, as these features do not capture the low-level features (e.g., orientation) that define these images. Conversely, retrieval accuracy is well above chance for complex stimuli, with vision retrieval predictably outperforming imagery retrieval. For the hidden layer ViT-L/14 embeddings, we see improvements across the board for all stimulus types relative to the pooled embeddings, reinforcing our conclusion from Section 4.2.6 that these sparser image embeddings provide a lot of utility in a retrieval context.

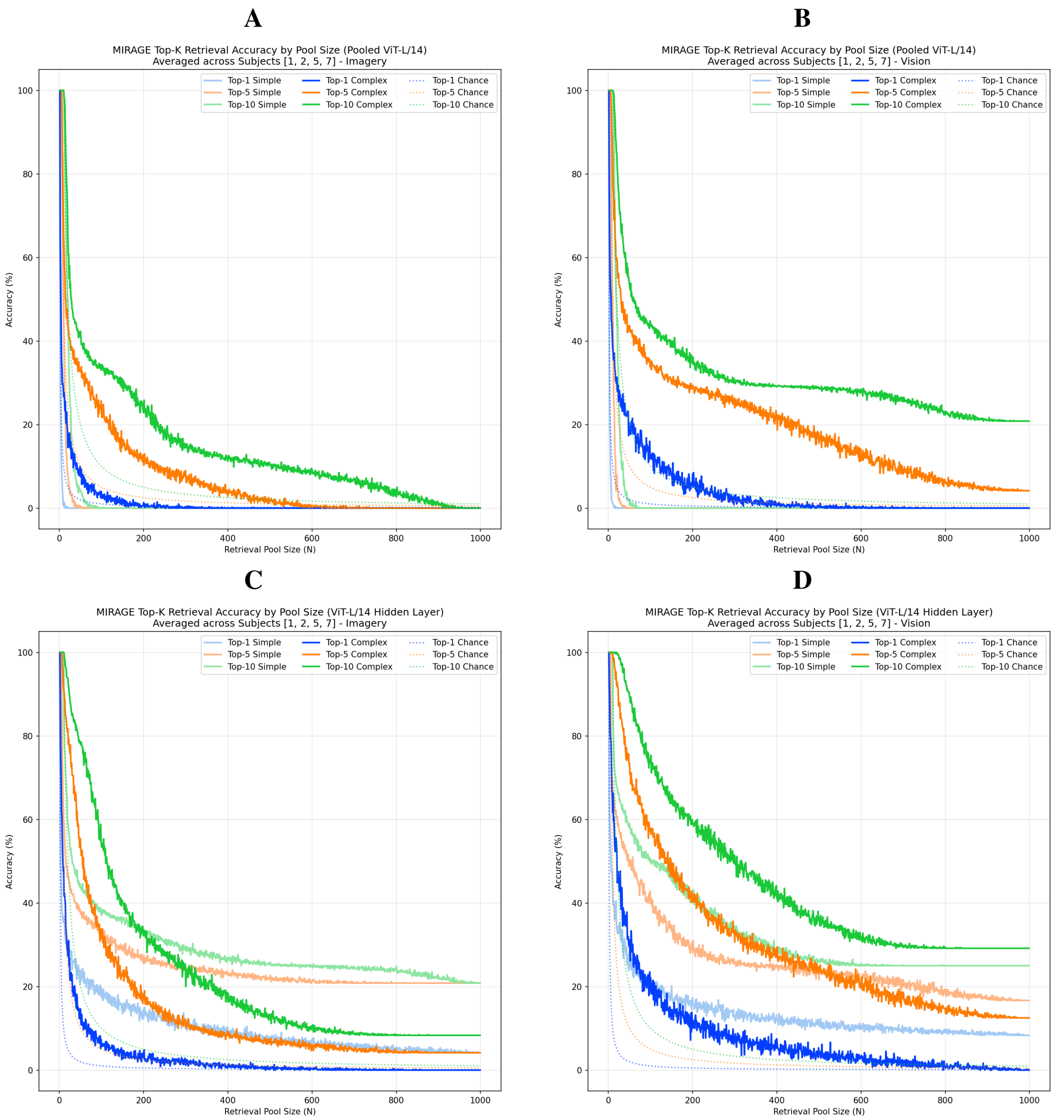

Figure Q: **Top-K retrieval performance vs. pool size for Subjects 1, 2, 5, and 7.** Accuracy (y-axis) is evaluated across varying distractor pool sizes (x-axis) for both mental imagery (left: **A, C**) and vision trials (right: **B, D**). The top row (**A, B**) evaluates retrieval in the pooled ViT-L/14 image embedding space used to drive the MIRAGE generative model, while the bottom row (**C, D**) uses the hidden layer ViT-L/14 space utilized in the retrieval pooling step (Section 4.2.6). Curves denote top-1, top-5, and top-10 performance for simple (light lines) and complex (dark lines) stimuli, with chance levels indicated by corresponding dotted lines. To calculate accuracy, the ground-truth NSD-Imagery stimulus is shuffled with N random distractor images from the NSD shared1000 pool; a success is recorded when the target image ranks within the top K closest matches to the subject's brain-predicted embedding. All curves are bootstrapped across 100 randomly sampled distractor pools for each value of N.

A.14 Behavioral experiment

A.14.1 Experiment protocols

We conducted a set of behavioral experiments on 500 human raters online. For our experiment, we identified no risks to the human participants, and our institution's IRB approved our experiment. We probed 3 experiments intermixed into two discrete sections within the same behavioral tasks, with each experiment consisting of trials sampled evenly from the 18 different stimuli, 3 stimulus types, and the 4 NSD subjects who completed all 40 scanning sessions (subjects 1, 2, 5, 7). After sampling

the target reconstruction, the distractor reconstruction was sampled from a pool of reconstructions from the same subject, stimulus type, and whether it was a vision/imagery reconstruction, but a different specific stimulus. The experimental trials within each task were shuffled and 36 trials were presented to each subject. Our subjects were recruited through the Prolific platform, with our experimental tasks hosted on Meadows. Each human rater was paid $1.50 for the completion of the experiment, and the median completion time was 6 minutes and 17 seconds, resulting in an average payment rate of $14.32/hour. Each human rater was presented with 6 attention check trials during the experiment. An attention check is a trial in which the ground truth image is presented as a candidate image during the trial. Because the ground truth image will always be the image that is most similar to itself, these trials were used to identify whether subjects were paying attention to the task and the instructions. We identified 5 human raters who failed at least 2 attention checks and removed those raters from our data before conducting our analysis. Raters were blind to the conditions of the experiment. The primary way we ensured consistency across trials was to keep the experiment very short (5m) and to sample a very large number of subjects (>500). Our goal with this design was to minimize any effects produced by the subject's rating profile changing over time as they saw more and more trials, and to minimize the biases of any one particular subject on the result of the experiment. Code to reproduce our experiment can be found in our anonymized GitHub repository. All human subjects provided written informed consent. All procedures were approved the Institutional Review Board at the University of Minnesota.

A.14.2 2AFC identification task

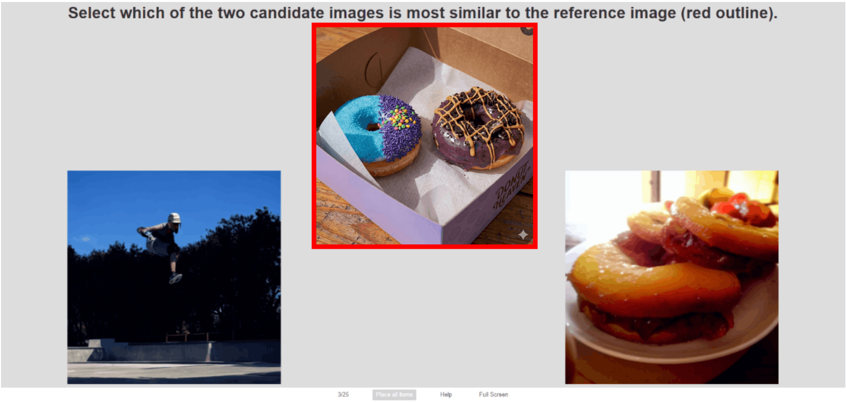

Figure R: An example of the 2 alternative forced choice task used in the first behavioral experiment performed by human raters.

Our first experiment, which made up the entirety of the first task, was a 2 alternative forced choice task (2AFC) facilitated by the "Match-To-Sample" task on the Meadows platform. An example of the first experiment can be seen in Fig R. In this experiment, human raters were asked to select which of two candidate images was more similar to a reference image. The reference image provided is the ground truth image the NSD-Imagery subject either saw or imagined, and the 2 candidate images were the target reconstruction of the reference image, or a randomly selected reconstruction from an fMRI scan corresponding to a different stimulus of the same stimulus type. The two candidate images were always sampled from the same reconstruction method and NSD-Imagery subject. This experiment was repeated for all reconstruction methods, visual modalities, NSD subjects, and across 10 reconstructions sampled from the output distribution of each reconstruction method. With the results presented in Section 2.2, we establish a baseline for human-rated image identification accuracy of mental image reconstructions, as no other paper has conducted behavioral evaluations of mental image reconstructions.

A.14.3 Continuous similarity rating task

The second and third experiments we conducted were shuffled together inside the second task of the experiment, which was facilitated by the "Drag-Rate" task on the Meadows platform. An example of the task used in experiments 2 and 3 can be seen in Fig S. In this task, human raters were presented

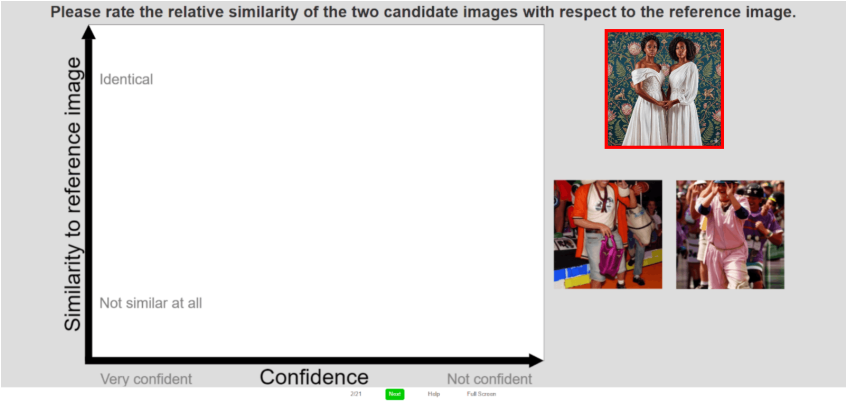

Figure S: An example of similarity score task used in experiments 2 and 3 of the behavioral experiment performed by human raters.

with a reference image, two candidate images, and a continuous two-dimensional plot that they could drag the candidate images onto, where the Y-axis represented "similarity to the reference image" and the X-axis represented the rater's confidence. The reference image provided was always the ground truth image the NSD-Imagery subject either saw or imagined. For experiment 2, the 2 candidate images were reconstructions of the reference image from the imagery and vision trials of the NSD-Imagery trials. Experiment 2 was repeated for the simple and complex stimuli (as conceptual stimuli do not have meaningful vision reconstructions), all reconstruction methods, NSD subjects, and across 10 reconstructions sampled from the output distribution of each reconstruction method. For experiment 3, we designed a head-to-head experiment for conceptual reconstructions, where the candidate images were reconstructions of the imagery trials for the conceptual stimuli produced by different reconstruction methods. Experiment 3 contained trials for all NSD subjects, 10 reconstructions sampled from the output distribution of each reconstruction method, and 3 unique combinations of reconstruction methods for each sample. One-dimensional similarity ratings—like the ones used in this section of the experiment—can be extremely sensitive to the context of the alternative samples being compared against, and so are primarily useful for comparing the relative similarity of the candidate stimuli presented during each individual trial. The two comparison tasks evaluated within this task of the experiment were designed with this in mind, each configured to more directly compare the difference in quality between reconstructions of vision and imagery, as well as to compare the differences in quality between reconstruction methods on the conceptual stimuli. Our analysis of these results in Section 2.2 provides a detailed analysis of how reconstruction performance scales across vision and imagery, and of how each method performs on the conceptual stimuli.

A.15 iCCN implementation

Originally introduced in Shen et al. [21], and first trained on NSD in Shirakawa et al. [51], we adapt the author's open source implementation to try and faithfully replicate their results, making the following changes to the implementation:

1. **Normalization of images:** We disabled normalization of images when computing VGG19 features. During our initial trials, normalization led to unexpected color distortions in the reconstructed images. Removing normalization allowed the reconstructions to maintain their original color integrity, which is particularly crucial for visual comparisons in tasks requiring precise color representation.
2. **Feature decoding with Ridge Regression:** Instead of the `fastl2lir` library, we employed the Ridge Regression implementation from the `sklearn` library. This change enhanced compatibility with the rest of our workflow and provided better support for managing memory-intensive computations. For VGG19 layers with a large feature space, feature decoding was performed in chunks. This approach enabled the simultaneous calculation of features and fitting of the Ridge Regression model without requiring intermediate results to be saved to disk, thereby optimizing both time and memory usage.

A.16 Public code release

We provide a public GitHub repository with the code to reproduce our method.

A.17 AI-Generated Images and Copyright Compliance

Several figures in this manuscript contain synthetic images generated by artificial intelligence. Specifically, the AI-generated panels within Fig 1, 2, 3, 4, 5, and B, C, D, E, F, G, H, I, J, K, L, O, R, and S in S1 Text were created using the Stable Cascade model developed by Stability AI.

In accordance with requirements for publishing under the Creative Commons Attribution 4.0 International (CC BY 4.0) license, we confirm that the use of these images complies with the software's terms of use. The Stability AI Non-Commercial Research Community License Agreement explicitly states under its definition of Derivative Works: "For clarity, Derivative Works do not include the output of any Model." Because the model creators do not claim copyright over the generated output, we, as the creators of these specific image outputs, license them under CC BY 4.0 for this publication.

Terms of Service Link: https://huggingface.co/stabilityai/stable-cascade/blob/main/LICENSE

In addition to the model outputs generated by the MIRAGE architecture, the "ground-truth" stimuli images for the complex stimuli containing natural scenes have also been replaced with AI-generated proxy images using Stable Cascade. The original experimental methodology utilized specific images from the Microsoft Common Objects in Context (MS COCO) dataset as visual stimuli during fMRI acquisition. While the annotations within the MS COCO dataset are open access, the underlying images retain the copyright of their original Flickr authors. Because several of the specific images used in our experimental subset carried restrictive licenses (e.g., NonCommercial, NoDerivatives, or All Rights Reserved) that are incompatible with open-access republication, we generated visually and semantically similar proxy images to serve as illustrative substitutes in the figures. We emphasize that these AI-generated proxy images shown in the manuscript are for illustrative purposes in the manuscript only. They are not the exact images shown to the human subjects during the fMRI data collection phase. All quantitative evaluations, fMRI-to-image model training, and performance metrics discussed in this paper were conducted using the original, exact MS COCO images. The substitution of these images in the published figures does not alter the underlying data, the MIRAGE model's architecture, or the reported quantitative results.
