# Supplementary material for: MIRAGE: Robust multi-modal architectures translate fMRI-to-image models from vision to mental imagery
Source: PLoS Comput Biol. 2026 May 22;22(5):e1014263. doi: 10.1371/journal.pcbi.1014263 (PMC13218618; doi:10.1371/journal.pcbi.1014263)
Supplement: Attachment — Submitted filename: Response to Reviewers.pdf [file pcbi.1014263.s002.pdf]

We would like to thank all reviewers for their feedback on our submitted manuscript. I was transitioning to a new position during the review period, and so we would also like to thank the reviewers for their patience as we prepared our response. We sincerely appreciate the feedback and opportunity to improve our contributions to PLOS Computational Biology.

Several reviewers identified issues with the availability of our data and code. We would like all reviewers to know that the link to the source code for our method has been updated and can be accessed in Appendix A.16 of our manuscript. The NSD-Imagery data used for this work has also been publicly released, and can be accessed at https://naturalscenesdataset.org/.

All line numbers and figure references refer to the updated "diff" version of our manuscript provided along with this letter, as these differ from the original submission in several places.

Replies to Reviewer #1.

> "*The authors state that their work is "a necessary step towards applications of mental image reconstruction, including diagnostic instruments for psychiatric conditions [63] and disorders of consciousness [64–66], and alternative communication methods for patients..". However, it is not immediately obvious to me why mental image reconstruction would be helpful in these cases. Why is it useful to be able to reconstruct an image specifically rather than decode the merely the concepts somebody is thinking about? Some discussion on exactly what the imagistic element would add would be helpful."*

We thank the reviewer for bringing up this important point of discussion. They are correct that significant communicative value would be provided by a "concept decoder" whose output is linguistic or otherwise compressed. However, visual representations of internal states could complement linguistic representations, for example by revealing how a specific concept or text prompt is visually interpreted. As the saying goes, 'a picture is worth a thousand words'. While our work makes no claims about being able to decode what is unique and idiosyncratic about a specific individual's mental imagery, it does represent a first step towards customized bespoke representations of internal states, and is a very literal attempt at this goal motivated by the overlap between representations of vision and mental imagery, and measured against a known ground truth visual stimulus image. We hope future work in this field will continue to advance the technology in this direction, providing more specific utility in the potential future use cases we identify in the paper. We have added a section to the manuscript encapsulating this point (lines 431-439)

> *"Relatedly, how viable is it to train these models in patients, given the large amount of data that is required? Adding some discussion on the practical implementations would be helpful."*

Thanks for raising this important point. The MIRAGE architecture we propose in this work does not make any significant advancements in this area, however there is a growing amount of interest in solving the "novel subject" problem in the area of fMRI decoding, and several works, including the MindEye2 paper [1] discussed in the manuscript, have managed to bring the necessary amount of fine-tuning data down to as little as one hour of scan time by pretraining on other subjects. While the MindEye2 fMRI-to-Image decoding model (like most)

tends to break down when applied to mental images, we believe to be only a matter of time until future research produces a method that is both robust to mental images, and is able to fine tune quickly on new subjects. We have added a discussion of this point on lines 461-468.

> *"I struggled with interpreting the similarity ratings between imagery and vision reconstructions. Why is it interesting to know whether participants rate the reconstructions as similar or not?"*

To be useful for applications, users, scientists, and clinicians will need to draw meaningful interpretations from the outputs of mental imagery decoding methods. Thus, human judgments of the quality of mental image reconstructions are important metrics of performance. Although the image feature metrics discussed in Section 4.1 may be a reasonable proxy for human judgment, numerous research efforts have established that these metrics do not always closely align with human subjective assessments of content [2] or quality [1,3], and a small dataset like NSD-Imagery can be particularly susceptible to their volatility. We have added a paragraph to the manuscript discussing this point (291-298). As for the relative similarity between reconstructions of seen and mental images shown in Figure 6B, we compared reconstructions in this way to get a specific sense of how different the reconstruction quality was between the two cases given that their ground truth visual content was identical. The slope of the line in Figure 6B thus provides us a way to measure the robustness of a particular method when applied to mental images, and is one of the specific analyses that highlights the success of MIRAGE in this generalization problem.

> *"For the influence of ablations, only qualitative comparisons in model performance are made. How can we be sure that decreases in performance are specifically due to these ablations and not due to e.g. randomness in the model initiation, reduction of complexity, or anything similar?"*

The ablation results visualized in Figure 7B are derived from the purely quantitative evaluation metrics as in Table 1. We noted in our analysis that changes in model initiation produced extremely small variations in model performance during our testing, and that the main source of variability is the stochasticity of the diffusion model used during the inference process, as mentioned in Section 4.1. To reduce the effect of this variability on the results, we output 10 reconstructions per test sample from each method and ablation, and report averaged metrics across them. This detail has been clarified in lines 259-261 of our submission, and we have added a visualization of the standard error of our ablation results in Figure 7B.

> *"What does the 'similarity score advantage' in experiment 3 mean exactly?"*

Thanks for the opportunity to clarify. Every trial in experiment 3 is a head to head comparison between two random reconstruction methods, and so over the course of the whole experiment, we gather lots of trials between all combinations of two methods. The "similarity score advantage" is the average difference between one method and another across these trials. For example, when MIRAGE and MindEye2 reconstructions are compared, MindEye2 reconstructions are rated on average as being 0.106 similar to the ground truth concept, while MIRAGE reconstructions are rated as 0.339 similar. The difference between these scores (0.233) is the "similarity score advantage" plotted in Figure 7A. This detail has been clarified in lines 344-347.

> *“It would be helpful to start the discussion section with a one or two sentence summary of the main question that was addressed.”*

We have added a new sentence to the discussion (lines 402-405) to clarify this point. Thank you for your thoughtful review of our work!

[1] Paul Steven Scotti, Mihir Tripathy, Cesar Torrico, Reese Kneeland, Tong Chen, Ashutosh Narang, Charan Santhirasegaran, Jonathan Xu, Thomas Naselaris, Kenneth A. Norman, and Tanishq Mathew Abraham. In Proceedings of the 41st International Conference on Machine Learning, 2024

[2] Pawan Sinha and Richard Russell. A perceptually based comparison of image similarity metrics. Perception, 40(11): 1269–1281, 2011. PMID: 22416586

[3] Yuval Kirstain, Adam Polyak, Uriel Singer, Shahbuland Matiana, Joe Penna, and Omer Levy. Pick-a-pic: An open dataset of user preferences for text-to-image generation. In Thirty seventh Conference on Neural Information Processing Systems, 2023.

Replies to Reviewer #2.

> *“Although the paper presents a well-constructed system, most components--ridge regression, CLIP embeddings, diffusion priors, and caption guidance--are known techniques. The main novelty lies in how they are combined and analyzed rather than in a fundamentally new algorithmic idea. The framing of the contribution should therefore emphasize empirical insight rather than architectural innovation.”*

We would like to thank the reviewer for the opportunity to clarify the contribution of our work. We agree that the improvements in mental image reconstruction performance arise from empirical insight. We have adjusted the manuscript (lines 56-58, 63) to make this framing more clear.

> *“Because diffusion models can strongly bias the reconstruction toward semantically plausible outputs, it’s unclear how much of MIRAGE’s performance actually reflects brain decoding rather than the model prior. The paper briefly acknowledges this in the appendix, but no systematic control experiments (e.g., with shuffled or random features) are provided. This issue is central to the scientific interpretation of the results.”*

We addressed this issue in two ways. First, in Experiment 1, the distractor image was a reconstruction decoded from a randomly selected brain activity pattern. Thus, both the "correct" and distractor reconstruction were shaped by the prior of the diffusion model. If this prior by itself were enough to produce accurate reconstructions, raters would have performed at chance; instead, their performance was well above chance. The experiment was thus designed to be a systematic control experiment on model performance, and we obtained p-values <0.001 for all

cases we evaluated, as reported in the caption of Figure 6A. We have clarified the description of Experiment 1 to make this more clear (lines 319-324)

Second, we performed a systematic analysis of the role of the prior in Appendix A.12 by varying the "strength parameter". When set to 0, the reconstructions are simply the "low-level" pictures obtained by decoding the VDVAE latents. These do not enjoy any guidance from the diffusion model. Interestingly, these reconstructions were **easier** to identify than high-strength reconstructions that are dominated by the guidance of the diffusion model and its prior, even though they suffered from low image quality. Thus, our results show that the prior of the diffusion model primarily enhances the image quality of the reconstructions, but not their identifiability. We have made this more clear in the revision on lines 388-399.

>*”The NSD-Imagery dataset is small (18 stimuli), which makes statistical confidence especially important. However, the paper reports only standard errors and no statistical tests or effect sizes. Similarly, human ratings are reported as averages without inter-rater reliability analyses. More rigorous statistics would substantially strengthen the empirical claims.”*

We thank the reviewer for the opportunity to strengthen our submission in this regard. The statistical significant tests for our human behavioral experiment were reported in the caption of Figure 6A (all $p < 0.001$). We now report them in the main text [line 324]. In addition, we now provide statistical tests in the caption for Figure 7A, and again find all $p < 0.001$ [line 349]

In lieu of an inter-rater reliability analysis, we chose a design focused on minimizing time-dependent changes in subject ratings and reducing individual subject bias. This was achieved by keeping the experiment extremely brief (approximately 5 minutes) and by sampling a very large number of subjects (over 500). We have clarified this in Appendix A.13.1 (lines 988-993).

> *“The ridge coefficient (λ=100,000) and the use of synthetic long captions (from LLaVA) are fixed design choices without justification of their robustness. Showing sensitivity analyses or ablations on these parameters would make the conclusions more solid.”*

The choice of ridge parameter value was based on the results of a hyperparameter tuning analysis we used to select the value of λ=100,000 in Appendix A.1. For the choice of synthetic captions, we provide an ablation analysis of performance using both a shorter format of synthetic captions, as well as the original captions provided as part of the COCO dataset in Figure 7B (discussed in lines 362-366). We have revised the manuscript to make these findings more clear in our submission (line 353).

> *“Several baselines are reproduced by the authors, while others use numbers reported in previous papers. It’s not fully clear whether all methods were evaluated under consistent conditions. Given that reconstruction quality can vary with sampling strategy or post-processing, this could bias the comparison.”*

We appreciate the opportunity to clarify this important part of our submission. All results reported in the paper were fully reproduced by us, and with consistent conditions. We took great care to ensure sampling strategies were as consistent as possible both with the original author’s

papers and with the other methods evaluated in our submission. We have updated the manuscript to make this important point more clear (lines 259-261).

> *“The authors suggest that text features help because higher-level brain regions in imagery are “language-like.” This is an interesting hypothesis but not directly supported by voxel-wise or region-based analyses. The paper would benefit from more evidence linking computational results to neural representations.”*

We thank the reviewer for the opportunity to clarify this point. Previous work has found that representations in higher-level visual cortex are language-like [1], but our point in the quoted text is an interpretation of an empirical result we observed, rather than a substantive scientific finding of our work directly linking the encoding of mental images in brain activity to a language-like representation. We have changed the writing to clarify the speculative nature of this hypothesis (lines 211-216 419-426), and look forward to future work studying this finding in more detail.

> *“While the ablation studies are good, the discussion of why certain factors help remains superficial. For instance, it would be useful to interpret the improvements from text guidance or reduced embedding dimensionality in light of known properties of visual or semantic cortex.”*

While our submission focuses primarily on the empirically effective strategies for improving reconstruction performance on mental images, we agree that the opportunity to interpret these mechanistic enhancements in the context of known properties of visual cortex is very appealing. In particular, we are not the first to find similar properties regarding the usefulness of language in describing activations of higher level visual cortex [1], and note that our findings support this previous work, despite it not being a focus of our submission. We have added a new subsection to the discussion that interprets our findings (lines 412-426), including the benefits of reduced dimensionality and text guidance, in the context of the low SNR of mental imagery and the semantic properties of higher-level visual cortex.

> *“The paper claims that the model "translates fMRI-to-image models from vision to mental imagery," yet it is unclear whether the observed success truly reflects cross-domain generalization or merely robustness to distribution shift. Since both training and testing are within the same dataset family (NSD and NSD-Imagery), a stronger demonstration on unseen subjects or imagery tasks would make this claim more convincing.”*

We appreciate the opportunity to further discuss this point. The core hypothesis of our work is that cross-domain generalization **is** a distribution shift, and thus that it can be addressed and overcome with the same techniques the field has used to overcome similar distribution shifts for decades, we believe the results of our work support this hypothesis, and thus make significant progress towards the goal of translating fMRI-to-Image models from vision to mental imagery.

With respect to unseen subjects, the task of adapting fMRI-to-Image models to novel subjects and datasets is far from a solved problem, and while recent work such as MindEye2 [2] has made progress in this regard, bringing the necessary fine-tuning data for a new subject from

40 hours down to 1 hour, we find empirically that MindEye2 and other multi-subject models tend to falter when applied to mental images. We have added a section of the manuscript discussing this point (lines 461-468), and we look forward to future work that will tackle this subject adaptation task in the context of mental imagery, but in the state of current literature, evaluating MIRAGE on unseen subjects is not possible.

We would also like to clarify that no NSD-Imagery data, and thus no mental imagery tasks whatsoever, were used in the training of MIRAGE. Because MIRAGE was trained exclusively on the vision trials of NSD core, the NSD-Imagery data and tasks *are* unseen tasks to the model during inference, and it is generalizing to this unseen task that is the core challenge tackled by our work. This detail has been clarified on lines 121-122.

> *"While comparisons are made against other deep neural decoders, there are no references to simpler baselines such as representational similarity mapping or direct retrieval from a large image-text database using fMRI features. This omission makes it difficult to assess whether MIRAGE's complexity is actually necessary."*

We have written extensively about the utility of retrieval methods as an approximation—or alternative—to reconstruction [3]. Retrieval and reconstruction are both valid approaches to image decoding. The main difference between them is the nature of the image prior. Retrieval relies on a static image corpus, effectively acting as a prior that assigns a non-zero, uniform probability to a finite set of candidate images and zero probability to all other possible images. In contrast, reconstruction utilizes a diffusion model that, in its sampling behavior, is more like a continuous probability distribution across the broader manifold of images it was trained on.

A systematic comparison between retrieval and reconstruction would be beyond the scope of the current work, as it would require extensive exploration of the interaction between the decoded feature space in which we measure distance to the retrieval candidates, and the size and content of the image corpus that supplies candidates for retrieval. Feature spaces that work well for reconstruction may not work well for retrieval, and vice versa, and this all depends upon the selection of the image corpus. We also think it is important to note that effective retrieval methods will not necessarily be less complex than the reconstruction pipeline of MIRAGE, since retrieval, like MIRAGE, incorporates either an encoding or decoding model and, ideally, a corpus of images that is as large as the corpus used to train the diffusion model used here.

However, we can meaningfully address the reviewer's important concern that the diffusion model used by MIRAGE may not actually be providing any value over retrieval, given the selection of feature spaces that MIRAGE deploys. The results of this new analysis can be found in Appendix A.13 of our revised manuscript (lines 941-969). We performed retrieval in CLIP space using a candidate corpus of 1000 COCO images sourced from the NSD shared1000 (a typical retrieval pool used in other NSD decoding papers). We show the top-1, top-5, and top-10 retrieval accuracy metrics as a function of the retrieval pool size for both seen and imagined simple and complex stimuli. We also compare the quality of the top retrieved image to the quality of the MIRAGE reconstruction in each case in Table 6. Across nearly all quantitative metrics evaluated, MIRAGE reconstructions outperform the top-1 retrieved image from the

1000-image COCO corpus on all stimulus types and for both vision and imagery. Thus, it is clear that appending a continuous diffusion prior to the end of the MIRAGE pipeline, rather than selecting likely approximations from a static image corpus, results in fundamentally superior, bespoke image decoding performance. Top-N metrics for retrieval accuracy are also well above chance for the seen and imagined complex stimuli, as expected, with the predictable result that vision retrieval is more accurate than imagery retrieval.

> *"The behavioral evaluation is impressive in scale but lacks transparency in design. The manuscript does not specify how stimuli were randomized, whether raters were blind to conditions, or how consistency was ensured across trials. Without these details, it is difficult to judge the robustness of the behavioral conclusions."*

We appreciate the opportunity to add clarity to our description of the behavioral experiment provided in Appendix A.14. Each trial during the experiment had a target reconstruction sampled from all 18 stimuli, 4 subjects, and the vision and imagery trials of the experiment. After sampling the target reconstruction, the distractor reconstruction was sampled from a pool of reconstructions from the same subject, stimulus type, and whether it was a vision/imagery reconstruction, but a *different* specific stimulus. Raters were blind to the conditions of the experiment. The primary way we ensured consistency across trials was to keep the experiment very short (~5m) and to sample a very large number of subjects (>500). Our goal with this design was to minimize any effects produced by the subject's rating profile changing over time as they saw more and more trials, and to minimize the biases of any one particular subject on the result of the experiment. We have revised Appendix A.14 to include these details and increase the clarity of our submission (lines 975-979, 988-993).

> *"Several metrics used (e.g., CLIP-based similarity) are inherently aligned with MIRAGE's feature space, since the model itself uses CLIP embeddings for decoding. This could give MIRAGE an advantage compared with baselines that use different representational spaces, potentially inflating performance estimates."*

We entirely agree that the use of a particular metric that is aligned with the feature space of the method being evaluated makes the metric less useful, it is for this reason that we report a wide variety of metrics, the vast majority of which are not aligned with the internal features of MIRAGE. This is also one of the core motivating factors behind our thorough behavioral experiments, as we desired an evaluation mechanism that was completely independent of any methodological choices of our method. We lastly note that with the exception of iCNN, all of the other methods we evaluate against in our paper also make use of a CLIP feature representation at some point in their method, and so MIRAGE will not obtain any meaningful benefit relative to the alternative methods as assessed by the CLIP metric in this regard.

> *"The discussion links MIRAGE's improvements to neurocognitive mechanisms of imagery (e.g., semantic and linguistic engagement). However, no direct neural evidence supports this explanation—no ROI-based correlation, temporal analysis, or representational similarity analysis is presented. These interpretations, while interesting, remain speculative."*

Please see response to Reviewer #1. We have revised the manuscript to clarify the speculative nature of our interpretations (lines 211-216, 419-426).

> *"The paper does not systematically discuss failure modes — for example, whether MIRAGE tends to hallucinate specific object types, blur boundaries, or misrepresent spatial layouts. Without such an analysis, readers cannot fully understand the model's limitations or potential biases."*

We did identify one salient failure mode, which is that stimuli that are out-of-distribution relative to images in the core NSD experiment (the training set) are poorly reconstructed. We added a new paragraph to our manuscript's discussion (lines 447-455) addressing this point. Additional analysis along the line suggested by the reviewer will require the development of innovative new evaluations. We think this is an important direction for the future but is beyond the scope of the current work.

> *"The abstract and introduction occasionally overstate originality. Phrases like "first robust multimodal architecture" could be rephrased as "a systematically evaluated multimodal framework for imagery decoding."*

We would like to clarify that the phrase "first robust multimodal architecture" does not appear anywhere in our manuscript, and we do not aim to make any such claims in our submission. With this in mind, we do appreciate the general suggestion to emphasize the strengths of our systematic and empirical work, rather than aspects of novelty or innovation, and have revised several sections of the manuscript accordingly (lines 55-59, 63, 402-406).

> *"Run a small-scale control where brain-decoded features are replaced with shuffled or averaged features, to quantify the prior's contribution."*

As discussed in a previous section, this was the primary goal and design of Experiment 1, our two-alternative forced choice behavioral experiment, which compared reconstructions of the stimulus with reconstructions generated with shuffled features. We have clarified this detail on lines 319-323.

> *"Show examples at different guidance strengths to illustrate when reconstructions are driven by brain signals versus prior bias."*

We appreciate this suggestion, and have added examples of reconstructions provided at different diffusion strengths to Appendix A.12 (Figure 22).

> *"Show how performance changes with λ and embedding size to confirm robustness."*

We provide an analysis of how performance changes with λ in Appendix A.1. Embedding size is a tricky thing to perform an ablation analysis over, as the embedding is fundamentally tied to the diffusion model used to do reconstructions. It is not possible to change the embedding size without also changing or retraining the diffusion model. We do provide an ablation analysis over different choices of diffusion model on lines 367-372, which validates our architectural choices.

> *“Indicate the number of samples averaged in each figure and ensure all key experimental details (e.g., λ grid search) are referenced in the main text.”*

We thank the reviewer for pointing out a few missing details and appendix references in our manuscript; we have fixed these specific cases in our revised manuscript (lines 259-261, 353).

[1] Aria Wang, Kendrick Kay, Thomas Naselaris, Michael Tarr, and Leila Wehbe. Better models of human high-level visual cortex emerge from natural language supervision with a large and diverse dataset. Nature Machine Intelligence, 5:1–12, 11 2023

[2] Paul Steven Scotti, Mihir Tripathy, Cesar Torrico, Reese Kneeland, Tong Chen, Ashutosh Narang, Charan Santhirasegaran, Jonathan Xu, Thomas Naselaris, Kenneth A. Norman, and Tanishq Mathew Abraham. In Proceedings of the 41st International Conference on Machine Learning, 2024

[3] Thomas Naselaris, Ryan J. Prenger, Kendrick N. Kay, Michael Oliver, and Jack L. Gallant. Bayesian reconstruction of natural images from human brain activity. Neuron, 63(6):902–915, 2009.

Replies to Reviewer #3.

> *“My main criticism with the paper is that it does not describe the dataset (and the protocol for recording it) in enough detail to ascertain whether the results are meaningful as brain decoding as opposed to representing something else (for example representing "time" as has been shown to be the case in studies with block design where training and testing items of the same class occur close in time).*

We thank the reviewer for raising this important point, and appreciate the opportunity to clarify the design of the NSD-Imagery experiment. While more complete details are provided in the CVPR paper referenced by the reviewer, the specific point about block designs and test/train overlaps is important and we have added a section to the manuscript (lines 96-129) describing it in more detail. Because NSD-Imagery was an extra session collected after the main NSD experiment, the session contains no trials used for training MIRAGE or any of the other models we evaluate in the paper. The design of the experiment splits the experiment by stimulus type, and presents the vision trials in a separate run before the imagery trials to ensure there are no confounds present from the visual response, and also ensure that the subjects had no problems remembering the stimuli they were instructed to imagine during the imagery trials. Because there are no training trials in any of the runs, there are no temporal relationships for the model to exploit during inference. While the main contribution of the manuscript is the MIRAGE method, and the database has been described elsewhere, we have added this new section to the submission describing the dataset in more detail to make these points more clear.

> *“details are missing in the processing steps of the algorithm. Authors say "we apply a set of image filters to boost the sharpness and contrast of the low-level images", but no detail of these filters are given. Please give all the details for this processing. What image filters do you use to "boost the sharpness and contrast of the low-level images" How are any hyperparameters in this process chosen?*

The specific filters we utilize are standard functions in the PIL.ImageEnhance library. In practice we did not explicitly optimize these hyperparameters, but picked reasonable default values that qualitatively produced the desired corrections to the low level images when testing our method on the held out NSD-Imagery subjects not used for evaluation, Subjects 3, 4, 6 and 8. These details, along with code to reproduce the filters, have been clarified on lines 835-843.

> *“The authors acknowledge that the standard metrics may not be reliable indicators of quality. Including human evaluations is great, but what about also looking at top-1 and top-5 retrieval accuracies?”*

Please see our response to Reviewer #2 above (“*While comparisons are made against….*”).

> *“How do you compute the "normalized feature metric performance" when some of the metrics are better larger and some better smaller. Please give the equation. Are median and worst case reconstructions based on this normalized metric?”*

To aggregate the feature metrics, we first standardized each metric S_k to the unit interval [0, 1]. For metrics where lower values indicated better performance (denoted by the set *L*), we applied an inverted Min-Max normalization such that the optimal raw value mapped to 1. The final score S_{final} was computed as the arithmetic mean of these normalized values, ensuring equal weighting across all image feature metrics:

$$\bar{S}_{\text{final}} = \frac{1}{K}\sum_{k=1}^{K}\left(\begin{cases}\frac{\max(S_k)-S_k}{\max(S_k)-\min(S_k)} & \text{if } k \in \mathcal{L} \\ \frac{S_k-\min(S_k)}{\max(S_k)-\min(S_k)} & \text{otherwise}\end{cases}\right) \tag{1}$$

Where:

- K is the total number of metrics.
- S_k denotes the raw score of the k-th metric.
- $\mathcal{L}$ is the set of metrics where a lower score indicates better performance.
- $\min(S_k)$ and $\max(S_k)$ are the minimum and maximum values of metric k across the dataset.

This normalized average of our image feature metrics was used to select the median and worst case reconstructions displayed in the paper. We have added a new section to the appendix (A.6.1, lines 872-884) describing this feature metric normalization process.

> *“While I agree that any experiments on patients and participants in general should be done with proper consent and IRB oversight, I do not think that fMRI recording should be defined as*

an invasive medical procedure. Any such statements should not be made lightly but by proper committees trained in these matters.”

Thank you for the opportunity to clarify our position. We agree that fMRI acquisition itself is physically non-invasive and has immense value in applications unrelated to decoding mental states. We have clarified the language on line 441 to reflect this.

Thanks again to all reviewers for their thoughtful review of our work, and for their patience during the peer review process.
