# Supplementary material for: MIRAGE: Robust multi-modal architectures translate fMRI-to-image models from vision to mental imagery
Source: PLoS Comput Biol. 2026 May 22;22(5):e1014263. doi: 10.1371/journal.pcbi.1014263 (PMC13218618; doi:10.1371/journal.pcbi.1014263)
Supplement: Attachment — Submitted filename: Response to Reviewers #2.pdf [file pcbi.1014263.s003.pdf]

Thank you to Reviewer #2 for their additional feedback, we have incorporated the suggested additional clarifications to our manuscript, in addition to re-organizing the main manuscript sections to be in alignment with journal requirements.

All section numbers, line numbers, and figure references refer to the newly updated "diff" version of our manuscript provided along with this letter, as these differ from the original submission and previous revisions in several places.

Replies to Reviewer #2.

> "1. In Section 6.2 (Societal Impact, lines 441–444), the manuscript states: "We propose that when deployed in a clinical setting, brain decoding should be defined as an invasive medical procedure…" In the response to Reviewer #3, the authors agreed that fMRI acquisition is physically non-invasive. These two statements are inconsistent. Please revise the main text, for example by removing the word "invasive" or rephrasing as "a medical procedure that yields private health information (even though fMRI acquisition itself is non-invasive)."

The word "invasive" was removed from line 435 in the previous revision to the manuscript, and does not occur anywhere in the current version of the paper.

> "2. In Section 5.3 and Appendix A.12, the authors state that "reconstructions with zero guidance (pure VDVAE decoding) remained highly identifiable." However, Figure 23 shows diffusion strength parameters only from 0.4 to 1.0, with no strength = 0 condition. Please clarify where the zero-guidance results are reported, or add a strength = 0 data point to the figure."

Thank you for bringing attention to this inconsistency, as noted on lines 1059-1060, the strength parameter has a nonlinear relationship to the amount of pixel-level variation in the output image, and below strength parameter values of 0.4, the image experiences no noticeable variation. It is for this reason that Figures 22 and 23 depict results between strength values of 0.4 and 1, and we have updated the discussion section to clarify that the results referenced in the claim do not use a strength parameter of zero. We have updated the Figure 22 caption and line 391 to clarify this point.

> "3. The response to Reviewer #1 regarding data-efficient fine-tuning (MindEye2) was thoughtful. Consider adding a forward-looking statement in Section 6.3 (Limitations), for example: "While current data-efficient models struggle with mental imagery, our results suggest that a linear backbone with multimodal guidance may be a promising direction for future subject-adaptive mental imagery decoding."

Thank you for the suggestion to improve this important point of discussion, we have added a statement in the Limitations section discussing this point on lines 461-462.

> "4. In Appendix A.13, the authors claim that MIRAGE "substantially outperforms both Top-1 retrieval baselines across the majority of quantitative metrics." While this holds for semantic and

high-level metrics, Table 6 shows that retrieval baselines are competitive or better on some low-level metrics (e.g., SSIM for complex stimuli). Please revise the conclusion to be more balanced, for example: “MIRAGE outperforms retrieval baselines on most high-level and semantic metrics, while retrieval remains competitive on certain low-level metrics.”

We have clarified the conclusion in this section (lines 1095-1097) to more accurately reflect the metric values in the analysis.
